# Green space exposure and Chinese residents’ physical activity participation: empirical evidence from a health geography perspective

**DOI:** 10.3389/fpubh.2024.1430706

**Published:** 2024-06-12

**Authors:** Qi-fei Xia, Guo-you Qin, Qi Liu, Yun-zhou Hu

**Affiliations:** ^1^Institute of Sports Training, Xi'an Institute of Physical Education, Xi'an, China; ^2^College of Physical Education, Hanjiang Normal University, Shiyan, China; ^3^College of Physical Education, Shaanxi Normal University, Xi'an, China; ^4^School of Athletic Performance, Shanghai University of Sport, Shanghai, China

**Keywords:** green space exposure, physical activity participation, NDVI, influence mechanism, healthy China

## Abstract

**Background:**

With continuous efforts made to promote the strategic goals of carbon neutrality and carbon peak, it is crucial to meet the growing and diversified needs of the public for fitness by practicing the concept of green development and promote the combination of national fitness and ecological civilization.

**Methods:**

To achieve this purpose, an OLS regression model was applied to estimate the role of green space exposure in Chinese residents’ participation in physical activity and its underlying mechanisms, using the microdata from the China General Social Survey (CGSS) data and the Provincial Vegetation Cover Index (NDVI) matched macrostatistical data.

**Results:**

The empirical results show that green space exposure significantly increases the probability of residents’ physical activity participation, and creating a green environment is conducive to creating a favorable physical activity environment for residents. Also, the core conclusions still hold after the year-by-year regression test is passed and the endogeneity problem is addressed. As revealed by mechanistic studies, green space exposure has indirect effects on the physical activity participation of residents through the independent mediating roles of reducing carbon emissions and promoting social interaction. According to heterogeneity results, males, those in marriage, and urban dweller groups are more inclined to perform physical activity in green spaces.

**Conclusion:**

The results show that the exposure of green space can help increase the probability of residents’ participation in physical exercise, and can that it achieved through two channels: reducing carbon emissions and enhancing social interaction. It is necessary to further strengthen the protection of the ecological lifestyle, give full play to the advantages of greenness and low-carbon, and create favorable conditions for the green development of a new model of national fitness.

## 1 Introduction

The World Health Organization (WHO) defines physical activity as any physical movement produced by skeletal muscles that requires the consumption of energy. Regular physical activity helps prevent and manage noncommunicable diseases such as heart disease and stroke (1). It is an important initiative to promote the modernization and development of sports in China by comprehensively promoting the construction of healthy China, accelerating the implementation of health actions and significantly improving people’s health. However, the increasingly fast pace of life continues to accelerate leads to the low participation rate of residents in physical exercise, and the health crisis caused by insufficient exercise threatens the physical health of the public. In 2018, the sports participation rate in China reached merely 30.9%, and Chinese residents spent an average of as little as 31 min participating in sports (2), posing a realistic challenge to the construction of healthy China. Since 2022, the State Council has successively issued the Opinions on Building a Higher Level of Public Service System for National Fitness, proposing to create a new green and convenient national fitness carrier, for the integration of national fitness with the construction of ecological civilization (3). As an important part of the natural ecosystem on earth, green space performs various functions in relation to the ecosystem, such as reducing noise and pollution, regulating hydrology, and purifying the environment (4). Moreover, it also has such positive effects as reducing obesity, relieving stress, and alleviating depression and anxiety (5). This kind of green space, which combines natural ecological and humanistic features, plays a role in promoting the participation of residents in physical exercise.

According to the 2022 National Fitness Trend Report, 74.3% of the respondents preferred to exercise outdoors, with the open spaces in squares, fitness paths, and garden paths and paved areas in parks as the main venues for fitness activities (6). With the increasing awareness of physical health among residents, outdoor sports and fitness exercises have been accepted as an important lifestyle for public entertainment and leisure. As urbanization and industrialization advance continuously at this stage, the expansion of urban land use has resulted in the destruction of ecological land use, the fragmentation of green space patterns, the reduction of biodiversity, air environmental pollution and a series of other ecological and environmental issues (7). Consequently, the demand for travel by residents is reduced. In contrast, there is a continuous increase in sedentary behavior, obesity rates and chronic disease mortality rates. Also, physical activity decreases (8). As an effective measure of green space exposure, vegetation is more often used to monitor the changes in regional ecological quality. It performs various natural functions required to create a favorable condition for leading the green movement and building a healthy life, such as enhancing the carbon sequestration effect (9), promoting photosynthesis (10), and releasing negative oxygen ions (11). As a parametric indicator, NDVI reflects the level of land vegetation cover according to remote sensing information, which allows the denseness and growth of vegetation to be quantified more accurately (12). Various green space attributes, including its type, size, quality and accessibility affect its social and ecological benefits (13). At present, there has been widespread recognition given to the health benefits created by green space exposure as a public activity space for residents’ physical exercise (14). Green space exposure affects resident physical activity participation in different aspects. On the one hand, according to the framework of natural ecosystem-ecological exposure-health effects, green space exposure improves health patterns through various mechanisms of action, such as the reduction of air pollution, heat, and noise, the reduction of mental and physiological stress among residents, and increased exercise and socialization (15). On the other hand, according to social ecological theory, individual behavior and development are embedded in the external environment, working together to contribute to individual health (16). Physical activity participation reduces the health burden imposed by transportation emissions, mitigates the regional heat island effect, and expands green space (17). However, developed countries such as Europe and the United States are the focus of most research on the association between green space exposure and physical activity. Besides, there are no scholars in China who have conducted empirical econometric studies on the relationship between the two. Does the fact that the residents of China, the world’s largest developing country, are in a green space-exposed environment have any effect on physical activity participation? Does this effect change over time? What is the specific mechanism of this effect? These are the questions to be explored in depth in this paper.

Therefore, an empirical study is conducted in this paper from the perspective of health geography using the China General Social Survey (CGSS) data and the Normalized Vegetation Cover Index (NDVI) matched data at the provincial level for two purposes. One is to examine the impact of green space exposure on residents’ participation in physical exercise and its mechanism. The other is to explore the heterogeneity of the direct mechanism in a differentiated way. In this way, empirical evidence and empirical support are provided to meet the diversified needs of mass fitness activities and to construct a green, convenient and universal fitness ecosystem.

## 2 Literature review

### 2.1 Measurement and temporal evolution analysis of green space exposure

In the existing studies, there is still no standardized way of defining green space exposure measurement. The common measures include two-dimensional spatial indicators such as green space rate, green space area per capita and service radius, as well as three-dimensional dimensions such as greening of urban roads, parks, squares, and other green volume measurements. Among them, the Normalized Vegetation Cover Index (NDVI) is the most effective measure of green space exposure oriented to population behavior and health (18). As a parameter used to evaluate ground greening, NDVI requires using the long time series of remote sensing data to monitor the change of vegetation cover systematically at the regional scale. In this way, the spatial and temporal situation of green vegetation cover can be intuitively reflected. Meanwhile, a specific formula is used to determine the value of the index. Finally, a value between −1 and + 1 is generated. When the value is closer to −1, it suggests that the ground is covered with more clouds, water, and snow; when the value is 0, it indicates that the surface is covered with rocks or bare soil, etc.; if the value is positive and closer to 1, it indicates that the ground vegetation cover is increasing. Based on the calculation of difference between the red and infrared bands (19), NIR is defined as the near-infrared light reflected by healthy vegetation. When the degree of vegetation cover rises, the red light reflection decreases and the near-infrared light reflection increases. It is calculated through the following formula:


$$\mathrm{NDVI}=\frac{\left(NIR-\mathrm{Red}\right)}{\left(NIR+\mathrm{Red}\right)}$$

**Figure 1:**
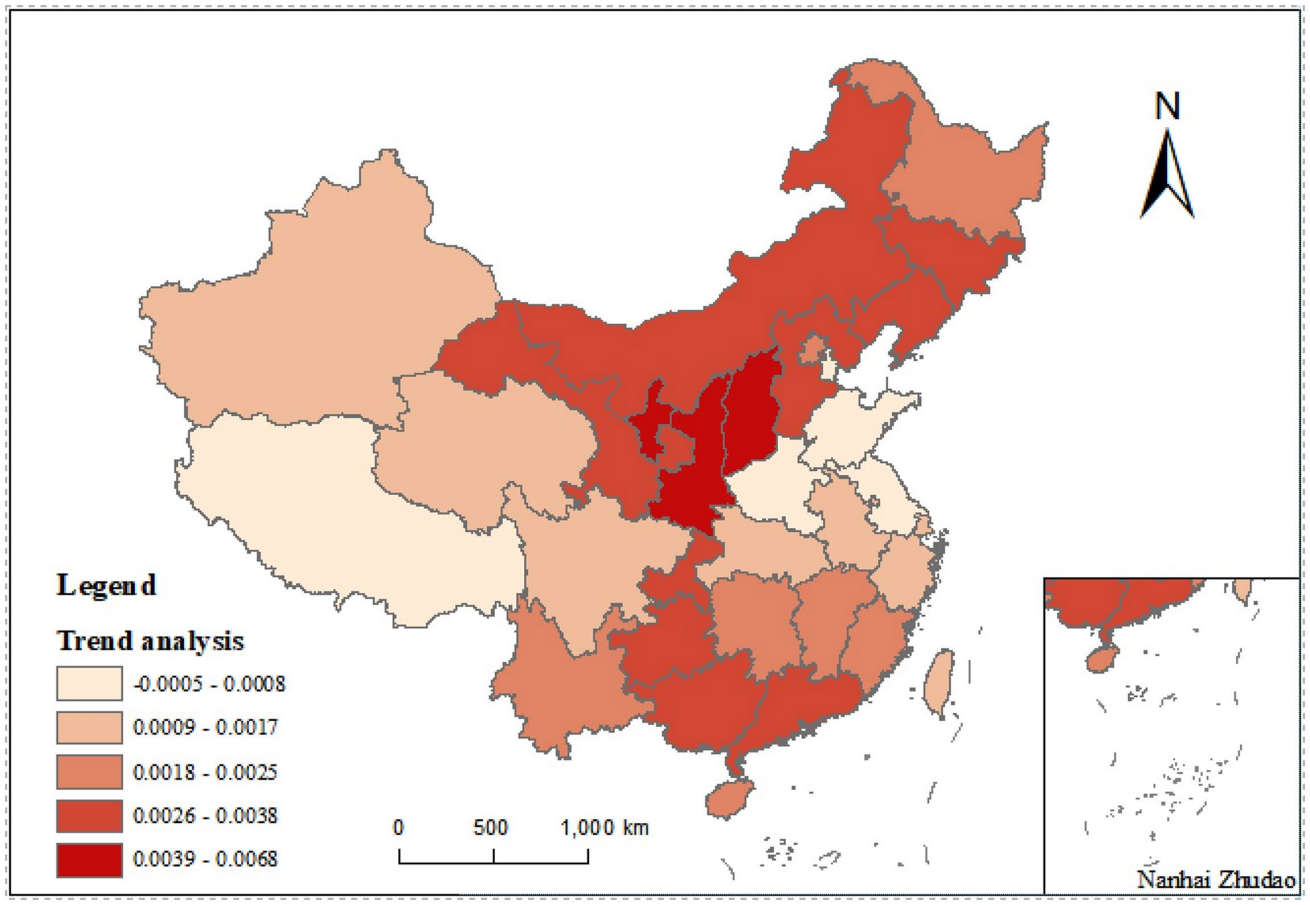
Characteristics and trend analysis of NDVI changes in Chinese provinces, 2000–2022.

In order to better analyze the dynamics of NDVI in China from 2000 to 2022, the Theil-Sen Median trend analysis and Mann-Kendall test (20, 21) were conducted to perform measurement. The Theil-Sen Median trend analysis is a non-parametric estimation technique commonly used to analyze longterm trends in vegetation NDVI (Normalized Difference Vegetation Index). Compared to other methods of trend analysis, it is less affected by outliers. Another nonparametric statistical test, namely the Mann-Kendall test, does not require the sample to follow a normal distribution. It is used to determine the significance of trends. By combining these two methods, the impact of climate change and human activities on vegetation growth can be better evaluated to reveal the longterm changes in NDVI. This approach is applicable to predict the effects of environmental factors on public sports. Considering SNDVI and Z-statistics, the trend of NDVI changes was delineated. Specifically, slope was delineated in the range of −0.0005 to 0.0005 for stable areas, no less than 0.0005 for the areas of improved vegetation, and less than −0.0005 for the areas of degraded vegetation. On the whole, the change of vegetation cover is crisscrossed; the degradation of vegetation cover is more significant in the eastern and western regions; the degree of vegetation cover shows improvement in the northwestern (including Shaanxi, Gansu, Ningxia, etc.), northeastern (Heilongjiang, Jilin, etc.) and northern (Beijing, Nei Mengu, etc.) regions; and the degree of vegetation cover in the southern region, as represented by Guangxi and Yunnan, is also improved significantly (Figure 1).

### 2.2 The green space effect: the direct effect of green space exposure on residents’ physical activity participation

In recent years, the continuous improvement of green infrastructure and social public sports service system prompts more and more scholars to explore how the transformation of national fitness can be promoted as a green ecological model, with the aim of making the public to receive more health benefits from green space exposure. According to the definition given by the U.S. National Environmental Protection Agency, in its Environmental Atlas, green space is referred to as all the areas covered by vegetation, including cropland, grasslands, woodlands, wetlands, and parks (22). Previously, the spatial effects of green space were assessed mainly according to land use types (23). However, green vegetation cover has now become a commonly used indicator of green space exposure for evaluation as satellite remote sensing technology advances (24). Through a review of the major practical advances on the health effects of green spaces at home and abroad, it can be found out that the focus of research is placed mainly on green space dose effects (25), the causal experiments on green space health in pathology (26), and restorative environmental gain effects (27). Specifically, there are some theories that can be applied to expound the impact of green space exposure on residents’ physical activity participation: (1) Social-ecological system theory. The American scholar Bronfenbrenner presented a systematic framework of socio-ecological models, arguing for the objective environment as a crucial influencing factor in health behavior interventions (16). As an important part of the natural environment, vegetation (including trees, green spaces, etc.) has positive effects on the physical and mental health of humans (15). As indicated in empirical research, a higher level of vegetation around the home or school may promote the physical or outdoor activity performed by humans (28). As revealed by Almanza et al., adolescents were 34–39% more likely to engage in physical activity in green spaces (29). (2) Self-determination theory. Proposed by American psychologists such as Deci, the motivational process theory of human self-determined behavior holds that individuals base their choices about their self-behavior on the full awareness of their personal needs and environmental information (30). Exercise adherence can be promoted when the enjoyment of exercise in a high-quality, pleasant green natural environment is intrinsically motivated (31). Those individuals engaging in green exercise regularly may understand the complementary benefits of green exercise compared to indoor environments either unconsciously or consciously, which reduces stress and invokes a sense of well-being (32, 33) (3) Space-behavior interaction theory. According to this theory, space (the geographic space of human activities) and behavior (human behavioral activities) are regarded as embodying a dialectical interaction and connection (34). Vegetation exhibits significant spatial variability properties. The residents living in high NDVI have relatively better somatic functioning and they are more likely to engage in regular physical activity within a green spatial environment, such as walking and calisthenics (35). In addition, those residents who exercise in more vegetated spaces experience less pollution, have more movable area, and engage in physical activity more frequently (36). Accordingly, Hypothesis H1 is proposed as follows:

*H*1: The higher the likelihood that residents exposed to green space will participate in physical activity.

### 2.3 Carbon sequestration: mechanisms of carbon emissions

Carbon emissions may be an important mechanism through which green space exposure exerts influence on residents physical activity participation. In a narrow sense, carbon emissions are defined as the emissions of “carbon dioxide” (CO_2_). Broadly speaking, it involves the emissions of six gaseous substances, including carbon dioxide (CO_2_), methane, nitrous oxide, oxyfluorocarbons, perfluorocarbons, and sulfur hexafluoride (37). In recent years, the carbon sequestration effect of vegetation has become the focus of research conducted by many scholars. In general, vegetation shows a high rate of photosynthesis, which increases the capacity to absorb CO_2_ (38). Also, carbon emissions can be indirectly reduced through the functions of green space such as containing water, regulating climate anomalies, and absorbing pollutants (39). As a significant means of maintaining ecosystems, the carbon sequestration capacity of vegetation can enhance soil organic carbon sequestration and optimize the environment of local areas (40), which leads to positive social and ecological impacts (41, 42). By reducing carbon emissions, global health can be promoted. Also, the reduction in GHG emissions mitigates the health risks associated with air pollution, meets the demand of humans for high-quality living environments, and increases the amount of physical activity in the population (43, 44). Additionally, there is a close correlation between the health benefits of physical activity and the potential risk of pollution from carbon emissions. On the one hand, the emissions of greenhouse gases from transportation can be reduced by physical exercise, as an effective way to realize a low-carbon life, and the participation in walking, cycling and other sports and leisure activities. Also, individuals can develop a low-carbon concept in their daily exercise (45). On the other hand, excessive carbon emissions are likely to pose various natural hazards such as air pollutant concentration (46), frequent occurrence of extreme weather (47), and increased ecological risks to land (48). Thus, the convenience and comfort of residents’ outdoor activities are affected. Overall, there has been a general consensus reached in the academic community on the contribution of spatial pattern of vegetation to carbon sequestration. Besides, reducing carbon emissions may have a positive effect on engaging residents in outdoor physical activity. Accordingly, Hypothesis H2 is proposed as follows:

*H*2: Expansion of green space reduces carbon emissions, which in turn promotes physical activity participation among residents.

### 2.4 Cohort effects: a mechanism for social interaction

Aside from the abovementioned influencing mechanisms, social interactions were one of the reasons why participants engaged in green exercise (49). As the most basic form of communication between individuals, socialization is a social activity in which people have interaction with each other and engage in material and spiritual exchanges under certain conditions. According to plenty of empirical studies conducted globally on the effects of green space exposure on residents’ social interactions, the social value of vegetation effects is reflected to a large extent in the provision of open and free social spaces and nature experiences for social capital accumulation (50, 51). In prior research, it has been discovered that social interaction plays an important “bridging” role between green space and health (52). Green spaces are regarded as the places for residents’ social gatherings and other recreational activities, such as parks and greenways (53), and the socializing in public green space attracts more groups to participate in neighborhood interactions. This leads to the interactive feelings and attitudes toward each other through shared feelings and experiences, which allows disadvantaged groups access to more social capital while improving cross-group relations (54–56). Regarding the relationship between social interactions and physical activity participation, there are many studies confirming the positive relationship between social interaction and physical activity participation, i.e., the increase in frequency of social interactions translates into the increase in individual physical activity behavior (57). High-quality social capital is inseparable from high-quality social interactions, including verbal communication, behavioral interactions, and emotional exchanges (58). Also, sport social capital is better at explaining the social connection of interpersonal interactions in sport (59). Regardless of individual social capital or collective social capital, it has a positive impact on residents’ physical activity behaviors (60, 61). According to studies, the natural environments with high green vegetation, such as parks and meadows, perform the practical service function of social interaction, attracting more peers to participate in exercise for improved health (62). Accordingly, Hypothesis H3 is proposed as follows:

*H*3: Expansion of green space enhances residents’ social interactions, which in turn promotes physical activity participation.

**Figure 2:**
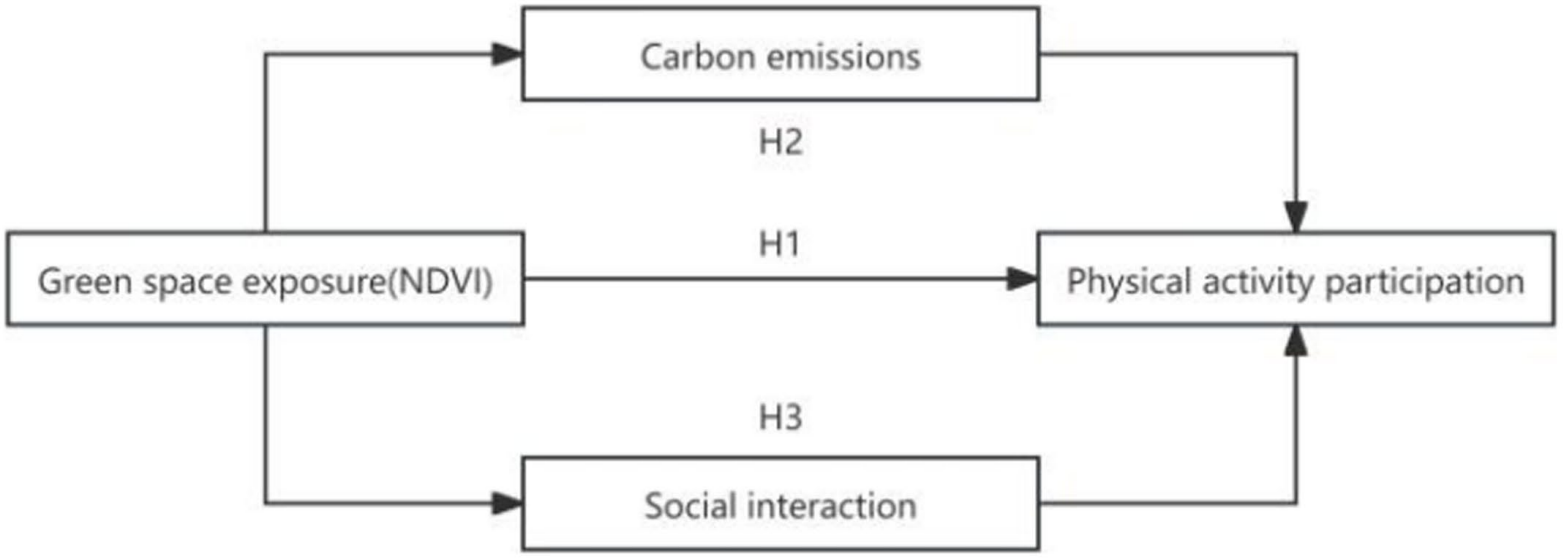
The theoretical framework.

By synthesizing the relationship argumentation and hypothesis derivation of the above relevant variables, we construct a theoretical framework model of green space exposure affecting residents’ participation in physical activity, and quantitatively assess the causal effect and mechanism of action. See Figure 2.

## 3 Materials and methods

### 3.1 Data sources

The population microsurvey data used in this paper is sourced from the China General Social Survey (CGSS), which is collected every two years, with the latest year updated to 2021. Covering 28 provinces (except Tibet, Hong Kong, Macao, and Taiwan), this data Research Topic reflects China’s social, economic, demographic, educational, and health changes, showing high representativeness and reliability. In this paper, China’s provincial NDVI macro data were matched with the micro China General Social Survey (CGSS), which is processed as follows. Firstly, the names of the required population-level variables were harmonized across the six periods of the CGSS database (2012, 2013, 2015, 2017, 2018, and 2021). Secondly, the microdata collected during a single year were longitudinally merged and then the merged data were cleaned. Again, the variables required for normalized vegetation index (NDVI) data for each province in China were calculated, extracted, and cleaned. Finally, the cleaned CGSS survey data were horizontally matched with the Normalized Vegetation Index (NDVI) using the CGSS province code and year code for urban and rural residents aged 18 years and older. In total, 40714 valid samples were obtained, with the sample size reaching 6415, 7297, 7081, 7481, 6882, and 5558 for the six surveys, respectively. As for the data on energy consumption of gasoline, coal and natural gas involved in the calculation of carbon emissions, they were collected from the China Economic Database (CEIO) and the China Energy Statistics Yearbook, etc., while the data on average annual precipitation in the provinces were sourced from the National Average Annual Precipitation Data by Province. All the results provided in this paper were calculated by Stata software.

### 3.2 Variable selection

#### 3.2.1 Dependent variable

Physical activity participation is a social activity aimed at developing physical fitness, enhancing physical health and enriching spare-time cultural activities. The dependent variable is the current participation in physical activity of the population, and the CGSS questioned the respondents about how often they had engaged in physical activity in the previous year, as measured by the questionnaire, “In the past year, did you often engage in the following activities in your free time - participating in physical activity?” In order to facilitate statistical analysis and understand the persistence of residents’ participation in physical activity, a reference was made to the existing study (63). The answers provided as optional include “never, a few times a year or less, a few times a month, a few times a week, and every day,” which are assigned a value of 1 ~ 5. The higher the value, the higher the degree of residents’ participation in physical activity, which is an ordered multicategorical variable.

#### 3.2.2 Independent variable

The independent variable used in this paper is the province normalized vegetation index (NDVI). In remote sensing images, the normalized vegetation index (NDVI) is taken as an important parameter that carries the information of vegetation cover changes. Herein, the NDVI dataset was preprocessed and calculated by using ENVI and ArcGIS software, etc. The value of NDVI ranges between −1 and 1. The closer the value is to 1, the higher the density of the green vegetation is (64).

#### 3.2.3 Mediating variables

In this paper, the mechanism of action was analyzed from two perspectives: carbon emissions and social interaction. Carbon emissions are calculated using IPCC and the methods proposed by the Office of the National Coordinating Group for Climate Change Response and the Energy Research Institute of the National Development and Reform Commission (NDRC). The formula of CO2 calculation is expressed as follows:


$$CO_{2}=\overset{7}{\underset{k=1}{\sum }}E_{K}*CF_{k}*CC_{k}*COF_{k}*44/12+m_{0}\cdot Q$$

In Equation, the CO2 emission coefficient consists of the energy consumption of seven fossil fuels, including coke, coal, kerosene, diesel, gasoline, combustion oil and natural gas. Such gases persist in the atmosphere for a longer period of time, accounting for a high percentage. Given the ease to statistically account for them, the total amount of CO2 emissions is taken as the mediating variable to perform logarithmic treatment.

The second type of mediator variable used in this paper is social interaction. As for CGSS data, the question to be raised is: “In the past year, have you frequently engaged in the following activities during your leisure time: visiting friends, relaxation, and educational pursuits?” The answers provided as optional include “never, seldom, sometimes, often, very often,” with a value of 1–5 assigned. The larger the value, the more frequent the social interaction.

#### 3.2.4 Control variables

The control variables used in this study were selected from other influencing factors in individual physical activity participation, including gender, age, household register, ethnicity, marital status, education level, health level, total personal income, and party member. Among them, gender is a dichotomous variable assigned a value of 1 for males and 0 for females. Age was defined as the year of the survey minus the year of birth. Household register was taken as a dichotomous variable, with urban areas assigned a value of 1 and rural areas assigned a value of 0. Ethnicity was assigned a value of 1 for Han Chinese and a value of 0 for other ethnic minorities (Mongols, Manchus, Hui, etc.). Marital status was categorized into not married and married. No marriage (unmarried, cohabiting, divorced, widowed) was assigned a value of 0, while being married (married for the first time with a spouse, remarried with a spouse, or separated but not divorced was assigned a value of 1). Education level was categorized as elementary school and below (without any education, private school and literacy class), junior high school, senior high school (general senior high school, vocational senior high school, middle school, technical school), university college (adult higher education, unified enrollment), and bachelor’s degree and above (adult higher education, unified enrollment, and postgraduate study). It was assigned the value of 1 to 5, respectively. For health level, from very unhealthy to very healthy, it was assigned a value ranging from 1 to 5. Total personal income is treated as natural logarithm. For party, party member was assigned a value of 1, and others were assigned a value of 0. In addition, control was imposed on province fixed effects and time fixed effects, considering the effect of time change and differences in economic development of respondents’ provincial regions. The details are presented in Table 1.

**Table 1:** Results of descriptive statistics.

Categories	Variables	Coding values	Mean	SD
Dependent variable	Physical activity participation	Never = 1, several times a year or less = 2, several times a month = 3, several times a week = 4, Everyday = 5	2.235	1.47
Independent variable	NDVI	Continuous variable	0.743	0.113
Mediating variables	Carbon emissions	Continuous variable	10.583	0.531
	Social interaction (such as visiting friends, relaxation, and educational pursuits)	Never = 1, rarely = 2, sometimes = 3, often = 4, very often = 5	2.738	0.988
Control variables	Gender	Female = 0, Male = 1	0.482	0.500
	Age	Age	55.293	16.341
	Ethnic	Other ethnic minorities = 0, Han Chinese = 1	0.916	0.277
	Party	Others = 0, Party member = 1	0.408	0.492
	Marital status	Not in marriage = 0, in marriage = 1	0.780	0.414
	Household register	Rural =0, 1 urban = 1	0.395	0.489
	Educational level	Elementary school and below = 1, middle school = 2, high school (vocational high school, junior college, technical school) = 3, college = 4, bachelor’s degree and above = 5	2.144	1.185
	Health level	Very unhealthy = 1, rather unhealthy = 2, average = 3, rather healthy = 4, very healthy = 5	3.575	1.085
	Total personal income	Logarithmic personal income	10.176	2.178
Instrumental variable	Average annual rainfall	Continuous variable	0.003	0.001

### 3.3 Research methods

#### 3.3.1 Baseline identification model

In this paper, Stata17.0 software was applied to analyze the relationship between green space exposure and residents’ participation in physical activity. Furthermore, the baseline regression model was established as follows by controlling province and time fixed effects:


(1) $$PA_{ijt}=\alpha +\beta_{1}NDVI_{ijt}+\delta C_{ijt}+\gamma_{t}+p_{j}+\mu_{ijt}$$

where *PA_ijt_* represents the physical activity participation status of individual residents of province *j* at time *t*; *NDVI_ijt_* denotes the change in the normalized vegetation index of individual province*_j_* at time*_t_*; *C_ijt_* represents a series of control variables affecting the physical activity participation of an individual, including age, gender, marital status, and education, etc.; *Y_t_* denotes the time-fixed effect; *P_j_* denotes the province-fixed effect; *α* indicates the constant term; *μ_ijt_* represents the error term; and *β_1_* and *δ* refer to the parameters to be estimated.

#### 3.3.2 Mechanism testing model

In order to verify the mediating role of carbon emissions and social interactions between green space exposure and residents’ participation in physical activity, the stepwise regression method proposed by Baron and Kenny was used in this paper to verify whether there is a mediating effect (65). The specific formula is expressed as follows:

(2) $$M_{ijt}=\alpha +\beta_{2}NDVI_{ijt}+\delta C_{ijt}+\gamma_{t}+p_{j}+\mu_{ijt}$$

(3) $$PA_{ijt}=\alpha +\beta_{3}NDVI_{ijt}+\theta M_{ijt}+\delta C_{ijt}+\gamma_{t}+p_{j}+\mu_{ijt}$$

In Equation (1), *β_1_* is the main effect, *β_3_* is the direct effect, *β2θ* is the indirect effect, and the relationship between the coefficients is expressed as *β_1_* = *β_3_* + *β_2_* × *θ*. Equation (2,3) *M_ijt_* is the mediator variable, including carbon emissions and social interactions. *β_2_*, *β_3_*, *θ* are the coefficients to be estimated, and *μ_ijt_* is the error term. The stepwise regression method is characterized by the low efficacy of statistical tests and the biased estimation of effects. In order to demonstrate the role of the mediating variables more accurately, the effects were decomposed using the “mixed effects” and “scale-change effects” decomposition method (KHB decomposition method) co-developed by Kristian et al. (66). Thus, the comparison in the independent contributions of the different mediating variables to the total effect was facilitated. This method is suitable when multidimensional mediating variables are involved.

## 4 Empirical results

### 4.1 Main characteristics of the survey sample

In order to better understand the changes in the main characteristics of the population in recent years, descriptive statistics were analyzed for the independent, dependent and mediating variables, etc., as shown in Table 2.

**Table 2:** Main characteristics of the survey sample.

Categories		Master sample	2012	2013	2015	2017	2018	2021
Master sample		40,714 (100%)	6,415 (15.76%)	7,297 (17.92%)	7,081 (17.39%)	7,481 (18.37%)	6,882 (16.9%)	5,558 (13.65%)
Physical activity participation	Never	20,222 (49.67%)	3,984 (62.1%)	4,407 (60.39%)	3,154 (44.54%)	3,662 (48.95%)	3,262 (47.4%)	1753 (31.54%)
	Several times a year or less	5,874 (14.43%)	935 (14.58%)	1,337 (18.32%)	1,101 (15.55%)	923 (12.34%)	880 (12.79%)	698 (12.56%)
	Several times a month	4,407 (10.82%)	597 (9.31%)	761 (10.43%)	897 (12.67%)	644 (8.61%)	681 (9.9%)	827 (14.88%)
	Several times a week	5,272 (12.95%)	481 (7.5%)	439 (6.02%)	886 (12.51%)	1,245 (16.64%)	1,249 (18.15%)	972 (17.49%)
	Everyday	4,959 (12.13%)	418 (6.52%)	353 (4.84%)	1,043 (14.73%)	1,007 (13.46%)	810 (11.77%)	1,308 (23.53%)
NDVI	Continuous variable							
Gender	Female	21,084 (51.79%)	3,856 (54.03%)	3,865 (52.97%)	3,667 (51.79%)	3,820 (51.06%)	3,538 (51.41%)	2,728 (49.08%)
	Male	19,630 (48.21%)	2,949 (45.97%)	3,432 (47.03%)	3,414 (48.21%)	3,661 (48.94%)	3,344 (48.59%)	2,830 (50.92%)
Age	Continuous variable							
Ethnic	Other ethnic minorities	3,406 (8.37%)	597 (9.31%)	739 (10.13%)	575 (8.12%)	641 (8.57%)	599 (8.7%)	255 (4.59%)
	Han Chinese	37,308 (91.63%)	5,818 (90.69%)	6,558 (89.87%)	6,506 (91.88%)	6,840 (91.43%)	6,283 (91.3%)	5,303 (95.41%)
Party	Others	24,095 (59.18%)	664 (10.35%)	702 (9.62%)	6,066 (85.67%)	6,406 (85.63%)	5,889 (85.57%)	4,368 (78.59%)
	Party member	16,619 (40.82%)	5,751 (89.65%)	6,595 (90.38%)	1,015 (14.33%)	1,075 (14.37%)	993 (14.43%)	1,190 (21.41%)
Marital status	Not in marriage	8,958 (22%)	1,241 (19.35%)	1,437 (19.69%)	1,385 (19.56%)	1705 (22.79%)	1,584 (23.02%)	1,606 (28.9%)
	In marriage	31,756 (78%)	5,174 (80.65%)	5,860 (80.31%)	5,696 (80.44%)	5,776 (77.21%)	5,298 (76.98%)	3,952 (71.1%)
Household register	Rural	24,641 (60.52%)	3,965 (61.81%)	4,668 (63.97%)	4,224 (59.65%)	4,562 (60.98%)	4,336 (63%)	2,886 (51.93%)
	Urban	16,073 (39.48%)	2,450 (38.19%)	2,629 (36.03%)	2,857 (40.35%)	2,919 (39.02%)	2,546 (37%)	2,672 (48.07%)
Educational level	Elementary school and Below	15,476 (38.01%)	2,613 (40.73%)	2,956 (40.51%)	2,761 (38.99%)	2,825 (37.76%)	2,776 (40.34%)	1,545 (27.8%)
	Middle school	11,855 (29.12%)	1908 (29.74%)	2,230 (30.56%)	2,144 (30.28%)	2,171 (29.02%)	1859 (27.01%)	1,543 (27.76%)
	High school	8,065 (19.81%)	1,275 (19.88%)	1,385 (18.98%)	1,346 (19.01%)	1,415 (18.91%)	1,313 (19.08%)	1,331 (23.95%)
	College	2,687 (6.6%)	397 (6.19%)	409 (5.61%)	424 (5.99%)	533 (7.12%)	457 (6.64%)	467 (8.4%)
	Bachelor’s degree and above	2,631 (6.46%)	222 (3.46%)	317 (4.34%)	406 (5.73%)	537 (7.18%)	477 (6.93%)	672 (12.9%)
Health level	Very unhealthy	1,502 (3.69%)	239 (3.73%)	236 (3.23%)	184 (2.6%)	346 (4.63%)	241 (3.5%)	256 (4.61%)
	Rather unhealthy	5,994 (14.72%)	1,089 (15.73%)	1,037 (14.21%)	988 (13.95%)	1,199 (16.03%)	1,114 (16.19%)	647 (11.64%)
	Average	9,298 (22.84%)	1,519 (23.68%)	1,430 (19.6%)	1,510 (21.32%)	1868 (24.97%)	1,409 (20.47%)	1,562 (28.1%)
	Rather healthy	15,414 (37.86%)	2,382 (37.13%)	2,675 (36.66%)	2,823 (39.87%)	2,708 (36.2%)	2,783 (40.44%)	2043 (36.76%)
	Very healthy	8,506 (20.89%)	1,266 (19.73%)	1919 (26.3%)	1,576 (22.26%)	1,360 (18.18%)	1,335 (19.4%)	1,050 (18.89%)
Total personal income	Continuous variable							
Carbon emissions	Continuous variable							
Social interactions	Never	3,869 (9.5%)	851 (13.27%)	438 (6%)	618 (8.73%)	624 (8.34%)	542 (7.88%)	796 (14.32%)
	Rarely	13,684 (33.61%)	2,104 (32.8%)	2,298 (31.49%)	2,230 (31.49%)	2,620 (35.02%)	2,459 (35.73%)	1973 (35.5%)
	Sometimes	13,711 (33.68%)	2,522 (39.31%)	2,580 (35.36%)	2,180 (30.79%)	2,517 (33.65%)	2,335 (33.93%)	1,577 (28.37%)
	Often	8,137 (19.99%)	809 (12.61%)	1702 (23.32%)	1,680 (23.98%)	1,524 (20.37%)	1,403 (20.39%)	1,001 (18.01%)
	Very often	1,313 (3.22%)	129 (2.01%)	279 (3.82%)	355 (5.01%)	196 (2.62%)	143 (2.08%)	211 (3.8%)

### 4.2 Baseline regression results

According to the research purpose and model setting of this paper, Hausman test was conducted to determine whether to use fixed or random model. According to the test results, it was significant at 0.05 level, rejecting the random effect hypothesis. Therefore, the fixed effect model was adopted. Multicollinearity was tested by measuring the variance inflation factor (VIF). It is generally accepted that there is a serious problem of multicollinearity if the VIF exceeds 2 for most of the variables, which affects the preestimation results of the study. As revealed by the measurement, the VIF values of all the variables ranged between 1 and 2, except for political status (2.15), and the Mean VIF value was 1.69. Since there was no serious problem of multicollinearity, it was considered suitable for regression analysis.

Table 3 lists the results of the baseline regression model, with the relationship between the effect of NDVI and residents’ participation in physical activity first explored. With control imposed on province and time fixed effects, the effect of NDVI on residents’ participation in physical activity was found to be positively and significantly correlated (*β* = 0.549, *p* < 0.01), suggesting a significant rise in the willingness of residents to participate in physical activity with an increase in the vegetation cover index. After the inclusion of control variables and province and time fixed effects, Model (2) yielded a positive coefficient on NDVI (*β* = 0.617, *p* < 0.01), and the coefficients on the control variables were all as expected. Specifically, the level of participation in physical activity is higher among the residents who are female, older adults, Han Chinese, party member, not in marriage, urban, having a high level of education, a high level of health, and a high total personal income. Since NDVI changes constantly from year to year, there may be obvious differences in the impact on residents’ participation in physical activity. Thus, independent regressions were performed on the data of six years, respectively, as a test on how the persistence and timeliness of NDVI impact residents’ participation in physical activity, so as to explore the impact of NDVI on residents’ participation in physical activity in more depth. According to the results of year-by-year regression, Models (3) to (8) all indicate that NDVI has a significant promoting effect on residents’ physical activity participation, and the increase was the highest in 2012. Through year-by-year regression, the impact of NDVI changes on residents’ participation in physical activity was verified, and evidence was provided to support the rationality of subsequent policy establishment and formulation. Thus, the conclusion was further confirmed.

**Table 3:** Baseline regression results of NDVI on physical activity participation of Chinese residents.

	Master sample	Master sample	2012	2013	2015	2017	2018	2021
Variables	Model (1)	Model (2)	Model (3)	Model (4)	Model (5)	Model (6)	Model (7)	Model (8)
NDVI	0.549***	0.617***	1.072***	0.687***	0.960***	0.359*	0.774***	0.458***
	(0.0661)	(0.0600)	(0.143)	(0.142)	(0.192)	(0.185)	(0.121)	(0.146)
Gender		−0.0311**	−0.0383	−0.0435*	0.0122	0.0422	0.0124	−0.0374
		(0.0128)	(0.0281)	(0.0236)	(0.0339)	(0.0320)	(0.0321)	(0.0397)
Age		0.00548***	0.000526	0.00143	0.00855***	0.00596***	0.00315***	0.00541***
		(0.000487)	(0.00109)	(0.000909)	(0.00127)	(0.00123)	(0.00121)	(0.00152)
Ethnic		0.136***	0.218***	0.0737*	0.102*	0.124**	0.251***	−0.0356
		(0.0232)	(0.0477)	(0.0389)	(0.0609)	(0.0571)	(0.0572)	(0.0948)
Party		0.134***	−0.467***	−0.365***	0.366***	0.401***	0.364***	0.252***
		(0.0189)	(0.0486)	(0.0423)	(0.0506)	(0.0493)	(0.0496)	(0.0531)
Marital status		−0.0335**	−0.143***	−0.134***	−0.0978**	0.00317	0.0218	0.0506
		(0.0155)	(0.0348)	(0.0293)	(0.0416)	(0.0379)	(0.0378)	(0.0447)
Household register		0.656***	0.613***	0.532***	0.560***	0.729***	0.711***	0.572***
		(0.0151)	(0.0333)	(0.0282)	(0.0402)	(0.0385)	(0.0383)	(0.0452)
Educational level		0.286***	0.256***	0.286***	0.215***	0.232***	0.268***	0.242***
		(0.00724)	(0.0174)	(0.0143)	(0.0194)	(0.0185)	(0.0184)	(0.0221)
Health level		0.105***	0.0744***	0.0360***	0.0870***	0.142***	0.105***	0.161***
		(0.00645)	(0.0140)	(0.0115)	(0.0169)	(0.0162)	(0.0162)	(0.0200)
Total personal income		0.0336***	0.00903	0.0516***	0.107***	0.0244***	0.0488***	−0.00172
		(0.00316)	(0.00575)	(0.00489)	(0.0160)	(0.00833)	(0.00840)	(0.00909)
Provincial fixed effects	Yes	Yes	Yes	Yes	Yes	Yes	Yes	Yes
Time fixed effects	Yes	Yes	Yes	Yes	Yes	Yes	Yes	Yes
Observations	40,714	40,714	6,415	7,297	7,081	7,481	6,882	5,558
R^2^	0.077	0.244	0.245	0.273	0.174	0.208	0.235	0.140

The parentheses are standard errors, ***, **, and * indicate significance at 1, 5, and 10% levels, respectively.

### 4.3 Robustness test

#### 4.3.1 Endogenous test

To prevent the endogeneity problems caused by omitted variables, DWH was used to test the presence of endogeneity problems. When the *F*-value is significant at the 1% confidence interval, it indicates an endogeneity problem of green space exposure affecting residents’ physical activity participation. The average annual precipitation was obtained from the average annual precipitation data of each province in China. To ensure the rationality of instrumental variable selection, there are 2 basic conditions to satisfy. One is the endogenous nature of the instrumental variables. Moisture is an important environmental factor that affects plant survival, growth and distribution. For smooth oxygenation and functioning, vegetation growth requires moderate moisture. The other one is the exogeneity of the instrumental variables. There is an absence of direct or indirect evidence that average annual precipitation has direct effects on the physical activity participation of the population. IV-2sls was used for estimation. According to the results, average annual precipitation had a significant effect on NDVI, and the F-statistic value exceeded 10%, indicating the absence of weak instrumental variable. Also, according to the principle of testing instrumental variables, there was no need to perform over-identification test when the number of instrumental variables equaled the number of endogenous variables. The second-stage regression of NDVI on residents’ participation in physical activity was significant at the 1% level, which was consistent with the results of the baseline regression. See Table 4.

**Table 4:** Endogeneity and robustness test.

Variables	First phase	Second phase	Replacement of empirical model	Change the independent variable
Average annual rainfall	40.149***			
	(0.459)			
NDVI		1.743***	0.790***	0.0763***
		(0.118)	(0.093)	(0.00936)
Control variables	Yes	Yes	Yes	Yes
Provincial fixed effects	Yes	Yes	Yes	Yes
Time fixed effects	Yes	Yes	Yes	Yes
DWH test	113.957***			
F-Statistical value	7642.59			
R^2^	0.352		0.100	0.242
Observations	40,714	40,714	40,714	40,714

The parentheses are standard errors, ***, **, and * indicate significance at 1, 5, and 10% levels, respectively.

#### 4.3.2 Replacement of empirical model

In order to make the empirical findings more reliable, an ordered probability model (Ologit) was employed to validate the findings. According to the results, with control applied on province and time fixed effects, NDVI had a positive effect on residents’ participation in physical activity and was significant at the 1% level, demonstrating the robustness of the findings. See Table 4.

#### 4.3.3 Change the independent variable

Through the equal interval classification performed in ArcGIS (67–69), the NDVI explanatory variables were categorized into five levels: Level I (0 ≤ NDVI ≤0.2), Level II (0.2 < NDVI ≤0.4), Level III (0.4 < NDVI ≤0.6), Level IV (0.6 < NDVI ≤0.8), and Level V (0.8 < NDVI ≤1.0). As indicated by the empirical results, the impact coefficient of NDVI was 0.0763 and significant at 1% level, which reaffirmed the robustness of the findings. See Table 4.

### 4.4 Mechanism of action analysis

According to the stepwise regression results, Model (2) shows that the coefficient of NDVI on carbon emission is −0.883, which is significant at 1% level, indicating a significant inhibitory effect of NDVI on carbon emission. In Model (3), physical activity participation as the outcome variable, NDVI as the predictor variable, and carbon emission as the mediator variable are factored into the regression equation, revealing that carbon emission has a significant inhibitory effect on residents’ physical activity participation. Thus, the mediator path of NDVI→carbon emission→physical activity participation is preliminarily verified. Model (4) shows that the coefficient of the effect of NDVI on social interaction is 0.272, which is significant at the 1% level. It indicates the significant contribution of NDVI to the social interaction of residents. According to the results of the mediation model of model (5), the coefficient of the impact of social interaction on residents’ participation in physical activity is 0.341, which is significant at the 1% level. Besides, the impact on participation in physical activity is less significant than the regression coefficient of the main effect of Model (1). It is initially judged that there is a mediation path of NDVI→social interaction→physical activity participation. See Table 5.

**Table 5:** Tests of mediating effects of carbon emissions and social interactions.

	(1)	(2)	(3)	(4)	(5)
Variables	Physical activity participation	Carbon emissions	Physical activity participation	Social interaction	Physical activity participation
NDVI	0.617***	−0.883***	0.474***	0.272***	0.525***
	(0.0600)	(0.0223)	(0.0610)	(0.0456)	(0.0580)
Carbon emissions			−0.162***		
			(0.0133)		
Social interaction					0.341***
					(0.00630)
Control variables	Yes	Yes	Yes	Yes	Yes
Provincial fixed effects	Yes	Yes	Yes	Yes	Yes
Time fixed effects	Yes	Yes	Yes	Yes	Yes
Observations	40,714	40,714	40,714	40,714	40,714
R^2^	0.244	0.198	0.246	0.035	0.295

The parentheses are standard errors, ***, **, and * indicate significance at 1, 5, and 10% levels, respectively.

In order to accurately demonstrate the independent role of the two mediating variables, carbon emissions and social interactions, the KHB decomposition method was used to decompose the mediating effects and further verify the robustness of the mediating roles. The KHB decomposition method performs better in clarifying the direct effect, indirect effect and the independent contribution of each mediating variable. According to Table 6, the mediating effects of carbon emission and social interaction account for 23.19 and 15.04% of the total effect, respectively, with neither of them including 0 at the upper and lower bounds of the 95% confidence intervals. It indicates that carbon emission and social interaction are the important mechanisms of action for the NDVI influencing residents’ participation in physical activity. See Table 6.

**Table 6:** KHB mediation effect decomposition test table.

	Carbon emissions			Social interaction		
Effect type	Coefficient	Lower limit	Upper limit	Coefficient	Lower limit	Upper limit
Total effect	0.617***	0.500	0.734	0.617***	0.500	0.734
Direct effect	0.474***	0.354	0.593	0.524***	0.411	0.638
Indirect effect	0.143***	0.119	0.167	0.092***	0.062	0.123
Indirect effect contribution rate	23.19%			15.04%		

The parentheses are standard errors, ***, **, and * indicate significance at 1, 5, and 10% levels, respectively.

### 4.5 Heterogeneity analysis

In order to reveal the differential impact of NDVI on the physical activity participation of residents with different characteristics, the sample was categorized into different gender, marital, and household cohorts for heterogeneity tests. According to Table 7, the gender cluster suggested that NDVI had a significant effect on physical activity participation for both male and female residents, with regression coefficients showing a more pronounced effect for male residents. As revealed by the marriage cohort, NDVI contributed more to physical activity participation among married residents. In terms of household cohorts, NDVI contributed more to physical activity participation among urban residents than rural residents, showing significance at the 1% level. The above results suggest a more important role of NDVI in physical activity participation for males, those in marriage, and urban residents.

**Table 7:** Heterogeneity of gender, marriage, and household register.

	(1)	(2)	(3)	(4)	(5)	(6)
Variables	Female	Male	Not in marriage	In marriage	Rural	Urban
NDVI	0.609***	0.639***	0.509***	0.651***	0.628***	0.638***
	(0.0842)	(0.0854)	(0.124)	(0.0685)	(0.0689)	(0.112)
Provincial fixed effects	Controlled	Controlled	Controlled	Controlled	Controlled	Controlled
Time fixed effects	Controlled	Controlled	Controlled	Controlled	Controlled	Controlled
Observations	21,084	19,630	8,958	31,756	24,641	16,073
R^2^	0.253	0.234	0.259	0.241	0.157	0.122

The parentheses are standard errors, ***, **, and * indicate significance at 1, 5, and 10% levels, respectively.

## 5 Discussion

In this study, six years of CGSS data were used to explore the correlation between green space exposure and physical activity participation among Chinese residents, and to examine whether an indirect effect is caused through mediating factors. This provides empirical evidence for promoting physical activity participation among Chinese residents. According to the above results, NDVI contributed significantly to the physical activity participation of Chinese residents, which is consistent with the results of previous studies. There is a significant increase in the likelihood of residents’ physical activity participation with a rise in NDVI. Also, this likelihood increases as the NDVI index changes. According to studies, the residents perceiving higher-quality greenery may spend more time participating in physical activity and experience better mental health, including urban parks, green belts, and streetscapes (70). However, there is also evidence suggesting no significant correlation between green space and physical activity (71–73). Similarly, in New Zealand, the access to parks is irrelevant to exercise (74). There are some possible reasons for this. One is that vegetation shows variation in growth between different regions of China due to precipitation and sunshine climate, and the other one is that the differences in green space attribute characteristics, quality characteristics, availability and accessibility all contribute to the differences in physical activity among residents. For example, Sallis et al. found out that the differences in park density caused variations in physical activity levels (75). Reportedly, a study conducted in Japan on the therapeutic effects of forests showed that the subjects scored significantly higher in subjective vitality when walking in the forest than in urban environments (76). According to a study, the edge density of urban vegetation fragmentation (e.g., scrubland) in the United States was associated with the active physical activity and normal BMI of residents. Also, a reduction in forest patch density may contribute to a higher prevalence (77). In another study, a different measure of green space exposure was used. Mytton et al. used GLUD data to conduct a cross-sectional survey and reported the relationship between green space and overall physical activity in the United Kingdom (78). It was found out that one in five residents living in green land in England had a lower mortality rate, which may be related to the engagement in more physical activities. However, the green space available to individuals in the environment was not considered to measure physical activity levels. With no control on some other factors, including weather and season, physical activity may be affected (79). Notably, the positive impact of green space exposure on physical activity is demonstrated by the fact that one study attempted to explore the potential mediating causal pathways between green space and health by constructing a mediated model of physical activity through a small-scale experimental study (28). In addition, attention shall be paid to the potential gaps in the use of parks and green spaces for vulnerable populations during the pandemic (80). Health agencies, including the American Public Health Association and the Centers for Disease Control and Prevention, have emphasized the importance of staying active while sheltering in place during COVID-19, such as visiting parks and green spaces (81). Proper physical distancing and face covering reduce the risk of infection, making parks and green spaces safe places for physical activity during the pandemic (82). This provides a reference for the government to develop a public health framework. In this study, the correlation between green space and Chinese residents’ participation in physical activity was explored using large-scale survey data. To a certain extent, this can refine the limitations of the results of the experimental causal study. However, this study ignored such factors as vegetation type, geomorphology, management, and maintenance, etc. Therefore, further studies will be carried out to empirically explore the relationship by collecting a wide range of availability data.

In addition, green space exposure can promote physical activity participation among residents by reducing carbon emissions. In previous studies, it has been found out that NDVI can improve population health by intercepting airborne particulate matter or by facilitating the uptake of gaseous pollutants through leaf stomata on the surface (83). In our study, it is shown that the carbon emissions from economic and social development can be offset by effectively utilizing the carbon sequestration function of vegetation, which creates a favorable ecological environment for residents’ participation in physical activity. Given a wide geographic area in China as covered by our study population, this result allows for the finding that increasing greenness and decreasing carbon emission pollution may have a synergistic effect on the health of the Chinese population. This makes it possible to promote physical activity. The greener the area, the more vegetation may be required to mitigate the negative impacts of atmospheric carbon dioxide emissions (84). The commonly proposed mechanisms include trees absorbing particulate matter such as oxygen-nitrogen compounds, sulfur dioxide, and ozone (85), with superior activity space created for residents to engage in physical activity (86). In addition to achieving carbon neutrality, building more green spaces plays an important role in maintaining climate-protected cities (87). Manchester, for example, has taken steps to expand green spaces by planting trees (88), which promotes low-carbon outdoor sports. Due to data availability and study time constraints, it was difficult to validate the differences in NDVI on carbon emissions and residents’ participation in physical activity in different provinces and regions of China. Allowing for this, it is necessary to conduct cluster analysis based on the geographic areas of green space exposure to understand the role of potential mechanisms more accurately.

Another finding of this study is that NDVI can promote physical activity participation by enhancing residents’ social interactions, which is consistent with existing research. The correlation between green space environments and social interactions has been demonstrated in numerous studies. For example, green environments can promote the normal interactions between communities (51), increase neighborhood tolerance (89) and reduce aggression (90). As revealed by further research, green space promotes the interaction between people, which in turn affects their health (91), and those with higher levels of participation in social interaction activities have better health (92). Compared to manmade built environments, high-quality green environments provide a high-quality natural experience, extend the time residents spend in the space or their willingness to visit, and increase the frequency and intensity of social interactions. Compared to those who are more isolated in their daily lives, the older adults with close social networks have been reported to walk in urban green space parks more frequently (93). At the same time, it is more likely for the residents with more social interactions to participate in physical activity. More importantly, social interaction has a potential to mediate the role between NDVI and physical activity participation. Parks and green spaces are the important places for residents to interact socially with others. As revealed by studies, adolescents value the opportunities to engage in physical activity and social interactions in urban green spaces, such as group exercise games. Overall, the green space plays a positive role in promoting the effective improvement of physical activity levels by creating an ideal and comfortable place for residents to engage in social interactions. Therefore, it is necessary to enhance ecological environmental protection, expand green development space, promote the construction of neighborhood social networks, and meet the residents’ daily exercise needs.

In our study, it was also found out that NDVI was more effective in promoting physical activity participation among men, those in marriage, and urban residents. From a gender perspective, males are more likely to participate in sport in green spaces, for two possible reasons. On the one hand, men and women differ in their lifestyles, environmental preferences and exercise habits. It has been shown that men who engage in instrumental physical activity are more likely to engage in the green movement (94). Men often engage in more outdoor activities and physical labor, which increases the likelihood of green space exposure. In comparison, women prefer walking to indoor places such as supermarkets and grocery stores. On the other hand, men and women tend to experience and utilize green space differently. When exercising in a park or greenway, women are more concerned about their personal safety (14) and they are less likely to engage in high-intensity physical activity (95). Notably, women may be more closely concerned with perceived health in green space environments despite the results suggesting that men have higher levels of exercise participation in green space. Also, the solidified notion of the division of family roles, which makes women assume more family caregiving responsibilities, may contribute significantly to the lower exercise participation of women. For detailed exploration in subsequent studies, applying control on home environment factors will be considered. In the marital cohort, NDVI was found to play a greater role in promoting physical activity participation among married residents. In terms of domicile groups, urban residents are more likely than rural residents to be physically active in green space. The variation in the carbon sink capacity of urban and rural green spaces is an important influencing factor for the differences in exercise among residents. As a strong guarantee for enhancing the carbon sink capacity of regional vegetation, sustainable urban development can not only increase the utilization rate of ecological units such as wetland parks, street greenspaces, pocket parks, etc., but also diversify outdoor sports facilities, thus significantly increasing the participation of urban residents in physical exercise.

The contributions of this study are as follows. Firstly, previous studies have focused more on developed countries (such as the United States, the United Kingdom or New Zealand), with a lack of attention paid to the empirical research on China as the largest developing country in the world. In this study, the relationship between the exposure to green space and residents’ participation in physical activity in China was analyzed to examine the two intermediary mechanisms of carbon emissions. This contributes to creating a good sports environment for the residents, thus promoting their active physical activity. Secondly, as physical inactivity is a major concern for global public health, a variety of factors were explored to analyze the demographic differences in the influence of green space exposure on residents’ participation in physical activity. This provides a guidance for local governments to formulate targeted policies aimed at improving the level of physical activities among residents. There are also some limitations in this study. Firstly, the data on population physical activity participation were collected using self-reports, which affected the ability to make causal inferences in this study. Secondly, to answer the question about whether the physical activity participation reported by respondents took place in a green space environment and the lack of investigation into the perceived aspects of green space environments, future research should be conducted to make causal inferences through the objective environmental perceptions of residents and quasi-experimental studies. Finally, due to the uneven distribution of green space across regions in China, the differential characteristics of exercise among residents of different provinces should be studied. Also, it is necessary to further quantify the effects of green vegetation types and spatial components on physical exercise.

## 6 Conclusion

With China General Survey Data (CGSS) and Provincial Vegetation Cover Index (NDVI) matched data as samples, the impact of green space exposure on Chinese residents’ participation in physical activity is examined in this paper from the perspective of health geography. Also, an empirical research is conducted on the mechanisms of carbon sequestration and cohort effect, which provides new theoretical perspectives and empirical evidences for accelerating the promotion of greening of lifestyles and the development of national fitness at a high level. According to the results, there is a green space effect of green space exposure on residents’ participation in physical activity, as manifested by the fact that an increase in NDVI promotes the active participation of an individual in physical activity. Notably, the conclusion still holds after robustness tests, such as endogeneity problem treatment, the replacement of the empirical model, and the change in the independent variables. As revealed by mechanistic analyses, carbon emissions and social interaction are the important mechanisms of action for green space exposure to influence residents’ participation in physical activity. According to heterogeneity analysis, group variability existed in green space effects. Comparatively, green space exposure has a more pronounced effect on physical activity participation among men, those in marriage, and urban residents.

## Data availability statement

Publicly available datasets were analyzed in this study. This data can be found at: http://cgss.ruc.edu.cn/ (accessed on 17 December 2023); the China vegetation index data (NDVI) can be found at http://www.gisrs.cn/ (accessed on 17 December 2023).

## Author contributions

Q-fX: Conceptualization, Data curation, Software, Validation, Writing – original draft. G-yQ: Formal analysis, Methodology, Writing – review & editing. QL: Project administration, Writing – review & editing. Y-zH: Supervision, Writing – review & editing.

## References

[1] World Health Organization. Physical Activity. Available at: https://www.who.int/news-room/fact-sheets/detail/physical-activity (Accessed on 29 May 2024)

[2] Statistics N.B.O. Statistical Bulletin of the People's Republic of China on National Economic and Social Development in 2020. People’s Daily. (2021). 10 p. doi: 10.28655/n.cnki.nrmrb.2021.002098

[3] Agency TXN. General Office of the CPC Central Committee and General Office of the State Council issued Opinions on Building a Higher Level of National Fitness Public Service System. Gazette of the State Council of the People's Republic of China. (2022) 10:8–12. Available at: https://kns.cnki.net/kcms2/article/abstract?v=KPcMkpFoYBDsxZGbYiruR9BEJjOLiD-dG0n_kD50PLzcY7JbrVktN4H3yCGetO64Pcs5w16NshdgyMsgjyjUTgbD4Q0xnFNrD7lxXMq-cGGTSLAV-cW44lscpTvf63WngTtDWulTftaHPPEj5cqogg==&uniplatform=NZKPT&language=CHS.

[4] Semeraro T Scarano A Buccolieri R Santino A Aarrevaara E. Planning of Urban Green Spaces: An Ecological Perspective on Human Benefits. Landscape. (2021) 10:25. doi: 10.3390/land10020105

[5] Amano T Butt I Peh KSH. The importance of green spaces to public health: a multi-continental analysis. Ecol Appl. (2018) 28:1473–80. doi: 10.1002/eap.1748, PMID: 30179305

[6] China, G.A.o.S.o. The "National Fitness Trend Report 2022" is released - Let's move together during National Fitness Week. (2022). Available at: https://www.sport.gov.cn/n20001280/n20001265/n20066978/c24565130/content.html (accessed on 2 April 2024).

[7] Haase D Nuissl H. Does urban sprawl drive changes in the water balance and policy? The case of Leipzig (Germany) 1870-2003. Landsc Urban Plan. (2007) 80:1–13. doi: 10.1016/j.landurbplan.2006.03.011

[8] Li FZ Liu Y Lü JJ Liang LC Harmer P. Ambient air pollution in China poses a multifaceted health threat to outdoor physical activity. J Epidemiol Community Health. (2015) 69:201–4. doi: 10.1136/jech-2014-203892, PMID: 24970766 PMC4514977

[9] Chen YZ Feng XM Tian HQ Wu XT Gao Z Feng Y. Accelerated increase in vegetation carbon sequestration in China after 2010: A turning point resulting from climate and human interaction. Glob Chang Biol. (2021) 27:5848–64. doi: 10.1111/gcb.15854, PMID: 34416063

[10] Gu L. Post W.M. Baldocchi D. Andy Black T. Verma S.B. Vesala T.; Wofsy S.C. Phenology of Vegetation Photosynthesis. In Phenology: An Integrative Environmental Science, Schwartz M.D., Ed.; Springer Netherlands: Dordrecht, (2003); pp. 467–485.

[11] Zhu SX Li JY He Q Qiu Q Su Y Lei T. Temporal Dynamics and Influencing Mechanism of Air Oxygen Content in Different Vegetation Types. Forests. (2024) 15:18. doi: 10.3390/f15030432

[12] Pettorelli N Vik JO Mysterud A Gaillard JM Tucker CJ Stenseth NC. Using the satellite-derived NDVI to assess ecological responses to environmental change. Trends Ecol Evol. (2005) 20:503-510. doi: 10.1016/j.tree.2005.05.011, PMID: 16701427

[13] Organization, W.H. Regional Office for Europe. (2016). Available at: https://iris.who.int/handle/10665/345751 (accessed on 3 April 2024).

[14] Cohen DA McKenzie TL Sehgal A Williamson S Golinelli D Lurie N. Contribution of public parks to physical activity. Am J Public Health. (2007) 97:509–14. doi: 10.2105/AJPH.2005.072447, PMID: 17267728 PMC1805017

[15] Markevych I Schoierer J Hartig T Chudnovsky A Hystad P Dzhambov AM. Exploring pathways linking greenspace to health: Theoretical and methodological guidance. Environ Res. (2017) 158:301–17. doi: 10.1016/j.envres.2017.06.028, PMID: 28672128

[16] Bronfenbenner U. The ecology of human developmentexperiments by nature and desing. Child Youth Serv Rev. (1979) 2:433–8.

[17] Nieuwenhuijsen MJ. New urban models for more sustainable, liveable and healthier cities post covid19; reducing air pollution, noise and heat island effects and increasing green space and physical activity. Environ Int. (2021) 157:8. doi: 10.1016/j.envint.2021.106850 PMC8457623 34531034

[18] Schipperijn J Bentsen P Troelsen J Toftager M Stigsdotter UK. Associations between physical activity and characteristics of urban green space. Urban For Urban Green. (2013) 12:109–16. doi: 10.1016/j.ufug.2012.12.002

[19] Tucker CJ. Red and photographic infrared linear combinations for monitoring vegetation. Remote Sens Environ. (1979) 8:127–50. doi: 10.1016/0034-4257(79)90013-0

[20] Li G Sun SB Han JC Yan JW Liu WB Wei Y. Impacts of Chinese Grain for Green program and climate change on vegetation in the Loess Plateau during 1982-2015. Sci Total Environ. (2019) 660:177–87. doi: 10.1016/j.scitotenv.2019.01.028, PMID: 30640086

[21] De Jong R de Bruin S de Wit A Schaepman ME Dent DL. Analysis of monotonic greening and browning trends from global NDVI time-series. Remote Sens Environ. (2011) 115:692–702. doi: 10.1016/j.rse.2010.10.011

[22] Pickard BR Daniel J Mehaffey M Jackson LE Neale A. EnviroAtlas: A new geospatial tool to foster ecosystem services science and resource management. Ecosyst Serv. (2015) 14:45–55. doi: 10.1016/j.ecoser.2015.04.005

[23] Vienneau D de Hoogh K Faeh D Kaufmann M Wunderli JM Röösli M. More than clean air and tranquillity: Residential green is independently associated with decreasing mortality. Environ Int. (2017) 108:176–84. doi: 10.1016/j.envint.2017.08.012, PMID: 28863390

[24] Klompmaker JO Hoek G Bloemsma LD Gehring U Strak M Wijga MH. Green space definition affects associations of green space with overweight and physical activity. Environ Res. (2018) 160:531–40. doi: 10.1016/j.envres.2017.10.027, PMID: 29106952

[25] Lee J Tsunetsugu Y Takayama N Park BJ Li Q Song CR. Influence of Forest Therapy on Cardiovascular Relaxation in Young Adults. Evid-based Complement Altern Med. (2014) 2014:7. doi: 10.1155/2014/834360 PMC3934621 24660018

[26] Hartig T Mitchell R de Vries S Frumkin H. Nature and health. Annual review of public health. (2014) 35:207–28. doi: 10.1146/annurev-publhealth-032013-182443 24387090

[27] Mackay GJ Neill JT. The effect of "green exercise" on state anxiety and the role of exercise duration, intensity, and greenness: A quasi-experimental study. Psychol Sport Exerc. (2010) 11:238–45. doi: 10.1016/j.psychsport.2010.01.002

[28] Lachowycz K Jones AP. Greenspace and obesity: a systematic review of the evidence. Obes Rev. (2011) 12:e183–9. doi: 10.1111/j.1467-789X.2010.00827.x, PMID: 21348919

[29] Almanza E Jerrett M Dunton G Seto E Pentz MA. A study of community design, greenness, and physical activity in children using satellite, GPS and accelerometer data. Health Place. (2012) 18:46–54. doi: 10.1016/j.healthplace.2011.09.003, PMID: 22243906 PMC3399710

[30] Dunn J.C. Zimmer C. Self-determination theory; Routledge: Abingdon, (2020); pp. 296–312.

[31] Baceviciene M Jankauskiene R. Self-Determined Motivation Mediates the Association between Self-Reported Availability of Green Spaces for Exercising and Physical Activity: An Explorative Study. Sustain For. (2021) 13:11. doi: 10.3390/su13031312

[32] Frühauf A Niedermeier M Elliott LR Ledochowski L Marksteiner J Kopp M. Acute effects of outdoor physical activity on affect and psychological well-being in depressed patients - A preliminary study. Ment Health Phys Act. (2016) 10:4–9. doi: 10.1016/j.mhpa.2016.02.002

[33] Coon JT Boddy K Stein K Whear R Barton J Depledge MH. Does Participating in Physical Activity in Outdoor Natural Environments Have a Greater Effect on Physical and Mental Wellbeing than Physical Activity Indoors? A Systematic Review Environ. Sci Technol. (2011) 45:1761–72. doi: 10.1021/es102947t, PMID: 21291246

[34] Zhang X Tang YF Chai YW. Spatiotemporal-Behavior-Based Microsegregation and Differentiated Community Ties of Residents with Different Types of Housing in Mixed-Housing Neighborhoods: A Case Study of Fuzhou. China Land. (2023) 12:23. doi: 10.3390/land12091654

[35] Gong Y Gallacher J Palmer S Fone D. Neighbourhood green space, physical function and participation in physical activities among elderly men: the Caerphilly Prospective study. Int J Behav Nutr Phys Act. (2014) 11:11. doi: 10.1186/1479-5868-11-40 24646136 PMC3994572

[36] Wang H Dai XL Wu JL Wu XY Nie X. Influence of urban green open space on residents' physical activity in China. BMC Public Health. (2019) 19:12. doi: 10.1186/s12889-019-7416-7 31409316 PMC6693084

[37] Liu Z. Carbon Emissions in China. In Carbon Emissions in China; Springer Theses-Recognizing Outstanding PhD Research; Springer-Verlag Berlin: Berlin, (2016); pp. 1–102.

[38] Wang SH Zhang YG Ju WM Chen JM Ciais P Cescatti A. Recent global decline of CO2fertilization effects on vegetation photosynthesis. Science. (2020) 370:1295. doi: 10.1126/science.abb7772, PMID: 33303610

[39] Guenther A. The contribution of reactive carbon emissions from vegetation to the carbon balance of terrestrial ecosystems. Chemosphere. (2002) 49:837–44. doi: 10.1016/S0045-6535(02)00384-3 12430661

[40] Jindal R Swallow B Kerr J. Forestry-based carbon sequestration projects in Africa: Potential benefits and challenges. Nat Res Forum. (2008) 32:116–30. doi: 10.1111/j.1477-8947.2008.00176.x

[41] Villa JA Bernal B. Carbon sequestration in wetlands, from science to practice: An overview of the biogeochemical process, measurement methods, and policy framework. Ecol Eng. (2018) 114:115–28. doi: 10.1016/j.ecoleng.2017.06.037

[42] Yang Y Tilman D Furey G Lehman C. Soil carbon sequestration accelerated by restoration of grassland biodiversity. Nat Commun. (2019) 10:7. doi: 10.1038/s41467-019-08636-w 30755614 PMC6372642

[43] Whitmee S Green R Phumaphi J Clark H Haines A. Bridging the evidence gap to achieve a healthy, net zero future. Lancet. (2021) 398:1551–3. doi: 10.1016/S0140-6736(21)02278-9, PMID: 34672966

[44] Haines A McMichael AJ Smith KR Roberts I Woodcock J Markandya A. Health and Climate Change 6 Public health benefits of strategies to reduce greenhouse-gas emissions: overview and implications for policy makers. Lancet. (2009) 374:2104–14. doi: 10.1016/S0140-6736(09)61759-1, PMID: 19942281

[45] Goodman A Brand C Ogilvie D Consortium IC. Associations of health, physical activity and weight status with motorised travel and transport carbon dioxide emissions: a cross-sectional, observational study. Environ Health. (2012) 11:10. doi: 10.1186/1476-069x-11-52 22862811 PMC3536622

[46] Shi XR Zheng YX Lei Y Xue WB Yan G Liu X. Air quality benefits of achieving carbon neutrality in China. Sci Total Environ. (2021) 795:9. doi: 10.1016/j.scitotenv.2021.148784 34246132

[47] Hansen J Kharecha P Sato M Masson-Delmotte V Ackerman F Beerling DJ. Assessing "Dangerous Climate Change": Required Reduction of Carbon Emissions to Protect Young People, Future Generations and Nature. PLoS One. (2013) 8:26. doi: 10.1371/journal.pone.0081648 PMC3849278 24312568

[48] Chen YZ Lu HW Li J Xia J. Effects of land use cover change on carbon emissions and ecosystem services in Chengyu urban agglomeration. China Stoch Environ Res Risk Assess. (2020) 34:1197–215. doi: 10.1007/s00477-020-01819-8

[49] White MP Elliott LR Taylor T Wheeler BW Spencer A Bone A. Recreational physical activity in natural environments and implications for health: A population based cross-sectional study in England. Prev Med. (2016) 91:383–8. doi: 10.1016/j.ypmed.2016.08.023, PMID: 27658650

[50] Gopal D Nagendra H. Vegetation in Bangalore's Slums: Boosting Livelihoods, Well-Being and Social Capital. Sustain For. (2014) 6:2459–73. doi: 10.3390/su6052459

[51] Krellenberg K Welz J Reyes-Päcke S. Urban green areas and their potential for social interaction A case study of a socio-economically mixed neighbourhood in Santiago de Chile. Habitat Int. (2014) 44:11–21. doi: 10.1016/j.habitatint.2014.04.004

[52] Maas J van Dillen SME Verheij RA Groenewegen PP. Social contacts as a possible mechanism behind the relation between green space and health. Health Place. (2009) 15:586–95. doi: 10.1016/j.healthplace.2008.09.006, PMID: 19022699

[53] Peters K Elands B Buijs A. Social interactions in urban parks: Stimulating social cohesion? Urban For Urban Green. (2010) 9:93–100. doi: 10.1016/j.ufug.2009.11.003

[54] Kazmierczak A. The contribution of local parks to neighbourhood social ties. Landsc Urban Plan. (2013) 109:31–44. doi: 10.1016/j.landurbplan.2012.05.007

[55] van den Berg P Arentze T Timmermans H. A multilevel analysis of factors influencing local social interaction. Transportation. (2015) 42:807–26. doi: 10.1007/s11116-015-9648-4

[56] Jennings V Bamkole O. The Relationship between Social Cohesion and Urban Green Space: An Avenue for Health Promotion. Int J Environ Res Public Health. (2019) 16:14. doi: 10.3390/ijerph16030452 PMC6388234 30720732

[57] McIntyre CW Watson D Cunningham AC. The effects of social-interaction, exercise, and test stress on positive and negative affect. Bull Psychon Soc. (1990) 28:141–3. doi: 10.3758/BF03333988

[58] Rojas H Shah DV Friedland LA. A Communicative Approach to Social Capital. J Commun. (2011) 61:689–712. doi: 10.1111/j.1460-2466.2011.01571.x

[59] Yanjie B Xiaolin LU. Theoretical Construction and Practical Significance of Sport Social Capital. J Shanghai University of Sport. (2022) 46:1–11. doi: 10.16099/j.sus.2021.12.26.0005

[60] McNeill LH Kreuter MW Subramanian SV. Social Environment and Physical activity: A review of concepts and evidence. Soc Sci Med. (2006) 63:1011–22. doi: 10.1016/j.socscimed.2006.03.012 16650513

[61] Hemingway JL. Leisure, social capital, and democratic citizenship. J Leis Res. (1999) 31:150–65. doi: 10.1080/00222216.1999.11949855

[62] Sugiyama T Leslie E Giles-Corti B Owen N. Associations of neighbourhood greenness with physical and mental health: do walking, social coherence and local social interaction explain the relationships? J Epidemiol Community Health. (2008) 62:e9. doi: 10.1136/jech.2007.064287 18431834

[63] Tao BL Chen HW Lu TC Yan J. The Effect of Physical Exercise and Internet Use on Youth Subjective Well-Being-The Mediating Role of Life Satisfaction and the Moderating Effect of Social Mentality. Int J Environ Res Public Health. (2022) 19:16. doi: 10.3390/ijerph191811201 PMC9517512 36141468

[64] NASA. NDVI. Available at: https://earthobservatory.nasa.gov (accessed on 15 April 2024).

[65] Baron RM Kenny DA. The moderator mediator variable distinction in social psychological-research-conceptual, strategic, and statistical considerations. J Pers Soc Psychol. (1986) 51:1173–82. doi: 10.1037/0022-3514.51.6.1173, PMID: 3806354

[66] Karlson KB Holm A Breen R. Comparing Regression Coefficients Between Same-sample Nested Models Using Logit and Probit: A New Method In: Liao TF, editor. Sociological Methodology, vol. 42. Newbury Pk: Sage Publications Inc (2012). 286–313.

[67] Zhang YR He Y Li YL Jia LP. Spatiotemporal variation and driving forces of NDVI from 1982 to 2015 in the Qinba Mountains. China Environ Sci Pollut Res. (2022) 29:52277–88. doi: 10.1007/s11356-022-19502-6, PMID: 35257346

[68] Zhu M Zhang JJ Zhu LQ. Article Title Variations in Growing Season NDVI and Its Sensitivity to Climate Change Responses to Green Development in Mountainous Areas. Front Environ Sci. (2021) 9:11. doi: 10.3389/fenvs.2021.678450

[69] Zhang Y Zhang LQ Wang JY Dong GC Wei YL. Quantitative analysis of NDVI driving factors based on the geographical detector model in the Chengdu-Chongqing region. China Ecol Indic. (2023) 155:14. doi: 10.1016/j.ecolind.2023.110978

[70] Sun PJ Song Y Lu W. Effect of Urban Green Space in the Hilly Environment on Physical Activity and Health Outcomes: Mediation Analysis on Multiple Greenery Measures. Landscape. (2022) 11:19. doi: 10.3390/land11050612

[71] Picavet HSJ Milder I Kruize H de Vries S Hermans T Wendel-Vos W. Greener living environment healthier people? Exploring green space, physical activity and health in the Doetinchem Cohort Study. Prev Med. (2016) 89:7–14. doi: 10.1016/j.ypmed.2016.04.021, PMID: 27154351

[72] Coombes E Jones AP Hillsdon M. The relationship of physical activity and overweight to objectively measured green space accessibility and use. Soc Sci Med. (2010) 70:816–22. doi: 10.1016/j.socscimed.2009.11.020, PMID: 20060635 PMC3759315

[73] Maas J Verheij RA Spreeuwenberg P Groenewegen PP. Physical activity as a possible mechanism behind the relationship between green space and health: A multilevel analysis. BMC Public Health. (2008) 8:13. doi: 10.1186/1471-2458-8-206 18544169 PMC2438348

[74] Witten K Hiscock R Pearce J Blakely T. Neighbourhood access to open spaces and the physical activity of residents: A national study. Prev Med. (2008) 47:299–303. doi: 10.1016/j.ypmed.2008.04.010, PMID: 18533242

[75] Sallis JF Cerin E Conway TL Adams MA Frank LD Pratt M. Physical activity in relation to urban environments in 14 cities worldwide: a cross-sectional study. Lancet. (2016) 387:2207–17. doi: 10.1016/S0140-6736(15)01284-2, PMID: 27045735 PMC10833440

[76] Takayama N Korpela K Lee J Morikawa T Tsunetsugu Y Park BJ. Emotional, Restorative and Vitalizing Effects of Forest and Urban Environments at Four Sites in Japan. Int J Environ Res Public Health. (2014) 11:7207–30. doi: 10.3390/ijerph110707207, PMID: 25029496 PMC4113871

[77] Tsai WL Floyd MF Leung YF McHale MR Reich BJ. Urban Vegetative Cover Fragmentation in the US Associations With Physical Activity and BMI. Am J Prev Med. (2016) 50:509–17. doi: 10.1016/j.amepre.2015.09.022, PMID: 26597506

[78] Mytton OT Townsend N Rutter H Foster C. Green space and physical activity: An observational study using Health Survey for England data. Health Place. (2012) 18:1034–41. doi: 10.1016/j.healthplace.2012.06.003, PMID: 22795498 PMC3444752

[79] Wolff D Fitzhugh EC. The Relationships between Weather-Related Factors and Daily Outdoor Physical Activity Counts on an Urban Greenway. Int J Environ Res Public Health. (2011) 8:579–89. doi: 10.3390/ijerph8020579, PMID: 21556205 PMC3084480

[80] Slater SJ Christiana RW Gustat J. Recommendations for Keeping Parks and Green Space Accessible for Mental and Physical Health During COVID-19 and Other Pandemics. Prev Chronic Dis. (2020) 17:5. doi: 10.5888/pcd17.200204 PMC7367064 32644919

[81] Centers for Disease Control and Prevention. Visiting parks and recreational facilities. (2019). Available at: https://www.cdc.gov/coronavirus/2019-ncov/daily-life-coping/visitors.html. [Accessed May 29 2020]

[82] Centers for Disease Control and Prevention. People who need to take extra precautions. (2019). Available at: https://www.cdc.gov/coronavirus/2019-ncov/need-extra-precautions/index.html. [Accessed May 29 2020]

[83] Ji JS Zhu AN Lv YB Shi XM. Interaction between residential greenness and air pollution mortality: analysis of the Chinese Longitudinal Healthy Longevity Survey. Lancet Planet Health. (2020) 4:E107–15. doi: 10.1016/S2542-5196(20)30027-9, PMID: 32220672 PMC7232951

[84] Shen YS Lung SCC. Mediation pathways and effects of green structures on respiratory mortality via reducing air pollution. Sci Rep. (2017) 7:9. doi: 10.1038/srep42854 28230108 PMC5322332

[85] Jim CY Chen WY. Assessing the ecosystem service of air pollutant removal by urban trees in Guangzhou (China). J Environ Manag. (2008) 88:665–76. doi: 10.1016/j.jenvman.2007.03.035, PMID: 17499909

[86] Wicker P. The carbon footprint of active sport participants. Sport Manag Rev. (2019) 22:513–26. doi: 10.1016/j.smr.2018.07.001

[87] Abu-Omar K Gelius P Messing S. Physical activity promotion in the age of climat e change [version 2; peer review: 2 approved]. F1000Research. (2020) 9:349. doi: 10.12688/f1000research.23764.2, PMID: 33628429 PMC7883311

[88] A Survey of Street Trees in Manchester, New Hampshire. (1998). Available at: https://extension.unh.edu/sites/default/files/migrated_unmanaged_files/Resource000405_Rep427.pdf. [Accessed May 29 2020].

[89] Cattell V Dines N Gesler W Curtis S. Mingling, observing, and lingering: Everyday public spaces and their implications for well-being and social relations. Health Place. (2008) 14:544–61. doi: 10.1016/j.healthplace.2007.10.007, PMID: 18083621

[90] Kuo FE Sullivan WC. Aggression and violence in the inner city - Effects of environment via mental fatigue. Environ Behav. (2001) 33:543–71. doi: 10.1177/00139160121973124

[91] Kaplan R. The nature of the view from home - Psychological benefits. Environ Behav. (2001) 33:507–42. doi: 10.1177/00139160121973115

[92] Haloi R Ingle NA Haloi E Kaur N Singh HP. Social Capital and Health: A Review. J Orofacial & Health Sci. (2015) 6:6. doi: 10.5958/2229-3264.2015.00025.8

[93] Enssle F Kabisch N. Urban green spaces for the social interaction, health and well-being of older people-An integrated view of urban ecosystem services and socio-environmental justice. Environ Sci Pol. (2020) 109:36–44. doi: 10.1016/j.envsci.2020.04.008

[94] Calogiuri G Elliott LR. Why Do People Exercise in Natural Environments? Norwegian Adults' Motives for Nature-, Gym-, and Sports-Based Exercise. Int J Environ Res Public Health. (2017) 14:15. doi: 10.3390/IJERPH14040377 PMC5409578 28375192

[95] Foster C Hillsdon M Thorogood M. Environmental perceptions and walking in English adults. J Epidemiol Community Health. (2004) 58:924–8. doi: 10.1136/jech.2003.014068, PMID: 15483308 PMC1732623

